# Monitoring disability inclusion: Setting a baseline for South Africa

**DOI:** 10.4102/ajod.v11i0.1020

**Published:** 2022-05-27

**Authors:** Helen Suich, Marguerite Schneider

**Affiliations:** 1 Independent Researcher, Canberra, Australia; 2 Department of Psychiatry and Mental Health, Alan J Flisher Centre for Public Mental Health, University of Cape Town, Cape Town, South Africa

**Keywords:** disability inclusion, South Africa, disability severity, psychosocial disability, monitoring, progressive realisation

## Abstract

**Background:**

The progressive realisation of disability inclusion requires political will and commitment, and comprehensive monitoring is necessary to give a clear understanding of what needs to be addressed and to highlight the gaps and barriers to the achievement of social inclusion of people with disabilities.

**Objectives:**

This article uses an existing dataset to present a baseline assessment of inclusion for men and women with no, mild or moderate disability severity and with no or moderate affect difficulties in South Africa.

**Methods:**

An existing dataset, capturing individual-level data for 8499 individuals, collected in 2019 across South Africa was analysed. Variables were utilised to represent aspects of nine domains of disability inclusion, and outcomes were compared using chi-squared tests (with Bonferroni adjustments) for groups categorised by disability severity and gender, and for affect severity and gender.

**Results:**

Overall, inclusion levels declined with increasing disability severity, and there were fewer differences in inclusion levels between those with and without affect difficulties than for those with functioning difficulties (as measured using the Washington Group on Disability Statistics’ Short Set of six questions on functioning).

**Conclusions:**

The article concludes by discussing several approaches to using the data to design policy responses, each of which results in a different range of domains that may initially be prioritised and targeted.

## Introduction

Inclusion, also referred to as social inclusion, is achieved when all people – including people with disabilities – have a sense of belonging to the ‘us’ of mainstream society rather than ‘them’, and of being recognised as a person being worthy of respect (Ikäheimo 2009:79). Realising disability inclusion requires political will and commitment, together with a clear understanding of what needs to be addressed in inclusion interventions.

The 2030 Agenda for Sustainable Development, following the framework provided by the United Nations Convention on the Rights of Persons with Disabilities (UN 2006) sets out the inclusion of people with disabilities as a significant contributor to reducing poverty and increasing growth, in addition to ensuring the rights and full participation of all, including people with disabilities (UN 2015). The Sustainable Development Goals encapsulate this with the inclusion of a number of indicators disaggregated by disability status so that progress towards inclusion can be monitored. The inclusion of people with disabilities in poverty reduction strategy papers also attests to the importance of inclusion in effective poverty reduction strategies (Adjei-Amoako 2016; MacLachlan et al. 2014; Mulumba 2011; Mwendwa, Murangira & Lang 2009; Wazakili et al. 2011). More narrowly, the business case for disability inclusion has also been clearly documented showing that a strong disability inclusive policy gives workplaces ‘access to talent, increased innovation, increased engagement and retention, a better reputation and benefits for everyone’ (ILO 2016:4).

Where inclusion goals or targets are set, monitoring should occur, incorporating the development of policies, their implementation and the outcomes of implementation, and encompassing both structural (van der Veen 2011) and individual perspectives (Schneider 2011) – to reflect how changes in infrastructure and systems impact on individuals with disabilities. Comprehensive monitoring of all of these aspects is necessary to highlight gaps and barriers that need to be addressed to achieve the social inclusion of people with disabilities. Ideally, such monitoring would be undertaken by the national statistics organisation (e.g. Statistics South Africa in South Africa), in an open and transparent manner so that organisations of people with disabilities and other support agencies trust the official figures. This trust would likely be strengthened if these organisations were also involved in selecting the detail of what was monitored.

Monitoring inclusion of disability at the policy level should examine the extent to which policies explicitly address disability issues within their development, and in the final document (Lang et al. 2019; Schneider et al. 2013). The lack of an explicit mention of disability inclusion will hamper implementation efforts to address the needs of people with disabilities. From a structural perspective, monitoring implementation processes assesses outcomes such as adherence to building accessibility regulations, implementation of inclusive education and employment practices and promotion of anti-discrimination awareness (van der Veen 2011). Individual-level outcomes can be monitored through individuals’ experiences and their perspectives on changing levels of participation across the range of relevant domains (Schneider 2011).

A rapid search was conducted on Scopus using the terms ‘disability AND inclusion’ and the first 150 papers (an arbitrary number that was deemed sufficient to give a reasonable sample) reviewed from the results yielded 116 relevant papers in total, published or in press in 2020 and 2021. Almost two-thirds of these papers were about inclusive education (e.g. Johora, Fleer & Hammer 2021; Kunz, Luder & Kassis 2021; Reese 2021), just in excess of one in 10 was about inclusion in employment and the workplace (e.g. Clube & Tennant 2021; Engeland et al. 2021), and the remainder (one-quarter) covered the legal aspects of inclusion (e.g. Buckley & Quinlivan 2021), or dealt with inclusion in religion (e.g. Bunga, Laure & Kiling 2021; Lorenzo & Duncan 2021), in disaster management (e.g. Alathur, Kottakkunnummal & Chetty 2021) and in community and physical spaces (e.g. Carnemolla, Robinson & Lay 2021; Cerdan Chiscano & Jiménez-Zarco 2021). This highlights the push at the global level for inclusive education for children with disabilities, and much less attention is given to other areas of inclusion. Only one study, by Santuzzi, Martinez and Keating (2021), addressed measurement issues and it argued that creating an inclusive work environment can facilitate disclosure and hence counting of the number of employees with and without disabilities. From this literature, it is not clearly evident which domains should be prioritised in promoting and achieving the progressive realisation of inclusion, or how to monitor this at the individual level.

In this article, we therefore focus on monitoring outcomes at the individual level across a range of inclusion domains, following a framework and ‘proof of concept’ of disability inclusion monitoring, described in the methods section and in detail in Schneider & Suich (2021). This framework is the basis for the data analysis presented, which examines the inclusion levels of individuals according to their disability severity, their anxiety and depression (or affect) severity and their gender. This analysis can, therefore, be viewed as a baseline assessment of inclusion for people with disabilities in South Africa, which can be used by policymakers and other stakeholders to identify and highlight priority areas for policy responses to improve inclusion. If future iterations of the same (or similar) data collection occurred, subsequent data analysis could then provide information about whether the progressive realisation of inclusion was being achieved, and in what domains.

## Research methods and study design

### The disability inclusion framework

The framework for the measurement and monitoring of key domains of disability inclusion was proposed in Schneider & Suich (2021), which also tested a proof of concept of disability inclusion measurement using an existing dataset. The original framework had 12 domains, selected through a literature review, and 40 indicators were selected for these domains on the basis of information available in the existing dataset, collected in South Africa in 2019 (described in detail in ‘The Individual Deprivation Measure South African data’ section below).

For the analysis presented in this article, the original framework has been modified – the number of domains has been reduced to nine, comprising 32 indicators, as shown in Table 1. The newly named ‘social relationships’ domain comprises the four (unchanged) indicators that were previously in the three domains of ‘interpersonal status’, ‘personal relationships’ and ‘being involved’ in the original framework. The change reduced the overlap of concepts being measured in these three domains, and improved the balance of the number of indicators in each domain across all domains. (Some imbalance remains, with the living conditions domain having 12 indicators, compared to a maximum of four in both the social relationships and personal safety domains.)

**TABLE 1:** Domains of inclusion and indicators used to measure aspects of those domains.

Domain of inclusion	Indicator
Social relationships	Unpaid domestic and care work humiliation
	Unpaid domestic and care work value
	Ability to reciprocate support received
	Community event inclusion
Living conditions	Food security
	Drinking water
	Domestic water
	Cooking energy
	Lighting energy
	Heating energy
	Home toilet facilities
	Toilet modifications
	Clothing and footwear ownership
	Clothing and footwear quality
	Bedding ownership
	Eviction concern
Economic opportunities and contributions	Labour force status
	Public transport availability and affordability
Support systems (formal and informal support)	Support availability
	Grant receipt
Institutional status	Identity document possession
	Birth certificate possession
Voice	Local decision-making inclusion
	Voting inclusion
Education	Schooling
	Basic literacy
	Basic numeracy
Healthcare access	Healthcare access and quality
Personal safety	Fuel collection hazards
	Water collection hazards
	Safety in the neighbourhood
	Safety at home

*Source:* Adapted from Schneider, M. & Suich, H 2021, ‘Measuring disability inclusion: feasibility of using existing multidimensional poverty data in South Africa’, *International Journal of Environmental Research and Public Health*, 18(9), 4431. https://doi.org/10.3390/ijerph18094431

Four indicators from the economic opportunities and contributions domain are not reported below, because there is insufficient information to determine whether each individual can be categorised as included or excluded on the basis of their activities (i.e. whether contributions were not made by respondents because they were unable, or because they were unnecessary). The four indicators were unpaid domestic and care work, fuel collection responsibility, water collection responsibility and on-call time. However, these four indicators – with relevant amendments to data collection tools – should be included in future, as they reflect a range and variety of productive contributions made by individuals to their household that should be recognised, irrespective of people’s disability status.

Two indicators in the support systems domain – receipt of a disability grant and receipt of an old-age pension – have been merged into a single indicator, ‘grant receipt’ as, in South Africa, each individual is potentially eligible to receive only one type of grant. One indicator (carer at home) in this domain is not reported, because the available information was insufficient for this analysis. The three indicators originally presented in the healthcare access domain have been merged into a single indicator, incorporating the information from all three of the original indicators for each individual.

### The Individual Deprivation Measure South Africa data

The data used in this analysis are a subset of the Individual Deprivation Measure (IDM) dataset for South Africa collected in 2019 and originally designed to measure multidimensional deprivation. The variables in the IDM dataset which best address aspects of the nine domains in the disability inclusion framework set out above have been utilised in this analysis, and are drawn from the main national sample. Comprehensive information about the data collection methods and the full dataset can be found in Schneider & Suich (2021) and Suich et al. (2020).

Enumeration areas (EAs) were randomly selected in each of the nine provinces of South Africa, stratified by rural or urban location, and all dwellings within the selected EAs were identified by using satellite imagery to remote sense roofs. Dwellings were then randomly sampled and approached for interview.

Data are presented for all eligible individuals interviewed – those aged 16 years and over residing in the sampled dwellings, who were able to give both informed and ongoing consent and were able to communicate for themselves, a total of 8499 individuals. Because no proxy interviewing was undertaken, the sample of people with disabilities is likely to be skewed to individuals with less severe disabilities, who were able to respond for themselves. All interviews were conducted face-to-face, using computer-assisted personal interviewing, in privacy, in any of the interviewees choice of the 11 official spoken languages of South Africa and by an enumerator of the same gender as the interviewee.

Data analysis was carried out using the R language (R Core Team 2021) in R Studio Version 1.4.1103 (R Studio Team 2020). The ‘survey’ package (Lumley 2020) was used to calculate the survey weighting and for the raking procedures, undertaken to maximise the generalisability of this sample to the national population using data on age, gender and race from the Community Survey 2016 (Statistics South Africa 2016). ‘Table one’ was used to produce relevant output for the analysis (Yoshida & Bartel 2020).

At both the indicator and domain levels, results are presented as the proportion of the weighted sample that falls into each of the three categories – inclusion, exclusion and in between – as described in detail below. In the comparison of outcomes, for disability severity and gender, and for affect severity and gender, exact *p*-values from chi-squared tests are reported, calculated using the Bonferroni adjustment for multiple comparisons to account for Type I errors (i.e. rejecting the hypothesis when it is true). Small differences between groups can be statistically significant with large samples such as this, so the results presented below focus on practical and meaningful differences between groups, not only those with statistically significant differences.

### Definitions of disability and affect severity

The measurement of disability in large-scale surveys has been consolidated through the work of the Washington Group on Disability Statistics (WG) in developing a set of questions about six core functioning domains referred to as the Short Set (WG SS) (WGDS ud) and which were included in the IDM survey tools. For disability severity, those classified as having no disabilities reported ‘no difficulty’ on any functioning domain, or ‘some difficulty’ on only one functioning domain, and those classified as having mild disabilities reported ‘some difficulty’ for between two and four functioning domains. Those classified as having moderate or severe disabilities (referred to as moderate severity below) reported ‘some difficulty’ for five or six functioning domains, or ‘a lot of difficulty’ or ‘cannot do at all’ for at least one functioning domain (or a combination of these responses). This includes the category five or more ‘some difficulty’ responses in the moderate category, but is otherwise the same as the recommended cut-offs for disability statistics reporting (Madans & Loeb 2017).

Mental health measures are important to include in any consideration of disability inclusion, as people with psychosocial disability are integral to the United Nations Convention on the Rights of People with Disabilities (UN 2006), and the WG is currently working on testing questions that identify people with such disabilities. Included in the WG Extended Set are questions on the frequency and severity of both depression and anxiety, which is known as the ‘affect’ domain (WGDS 2011). The WG has proposed that these four questions are combined to create an indicator of the presence and severity of affect problems or difficulties. Given the growing recognition of the importance of including people with psychosocial disability in monitoring disability, the affect indicator is used in this analysis as a way, albeit a limited one, to identify the seventh domain of disability in addition to the six domains in the WG SS.

Ongoing and unpublished work by the WG, on developing measures that ensure inclusion of people with psychosocial disability in disability statistics suggests that people with psychosocial disability are more likely to report more severe affect difficulties than people without (see e.g., Schneider & De Palma 2020). As such, the affect indicator in this analysis is reported with two levels of severity – none and moderate. Those reporting mild psychosocial disability have been merged into the ‘none’ category in a crude attempt to distinguish those with a psychosocial disability from those likely to be reporting distress associated with the impact of having a disability or in reaction to adverse life circumstances.

Table 2 shows the intersection of disability and affect severity in the sample, and the relative sample sizes and demographic characteristics for both groups can be seen in Table 3.

**TABLE 2:** Proportion of sample reporting disability severity and affect severity (%, unweighted).

Disability severity	Affect severity	
	None	Moderate
None	66.6	13.7
Mild	6.8	2.7
Moderate	6.8	3.5

**Total no.**	**6,816**	**1,683**

*Source*: IDM South Africa data, prepared by Suich and Schneider

**TABLE 3:** Demographic characteristics by disability severity and affect severity (%, weighted sample).

Characteristics	Disability severity			Affect severity	
	None	Mild	Moderate	None	Moderate
Original sample size (No.)	6,827	802	870	6,816	1,683
%	80.3	9.5	10.3	80.2	19.8
**Age**					
16–24	29.0	10.9	8.8	27.6	20.1
25–64	66.1	56.2	58.4	63.4	72.1
65+	4.9	32.9	32.8	9.0	7.8
**Gender**					
Male	49.7	34.7	30.6	48.5	41.7
Female	50.3	65.3	69.4	51.5	58.3
**Population group**					
Black African	77.4	80.0	80.3	76.7	83.0
Coloured	8.9	10.1	10.9	8.8	10.9
Indian or Asian	2.7	3.9	3.2	3.2	1.2
White	11.0	6.0	5.6	11.4	4.9

*Source*: IDM South Africa data, prepared by Suich and Schneider

Table 2 shows the intersection of the experiences of individuals with respect to functioning difficulties and anxiety and depression, with approximately double the proportion experiencing moderate affect but no disability compared to those with a moderate disability but no affect difficulties, and a relatively small proportion of the overall sample experiencing both moderate disability and moderate affect problems.

It is notable that in Table 3, there is a high proportion of women and older people (65 years and older) in both the mild and moderate disability severity categories, which is in line with global trends (Mitra & Sambamoorthi 2014), and shows relatively higher levels of moderate affect problems amongst women compared to men (see also Herman et al. 2009).

### Data analysis

#### Indicators

As noted above, 32 indicators were created from variables in the IDM dataset to measure different aspects of the nine domains of inclusion in the framework. For these indicators, each individual’s response was categorised into one of the three groups according to whether they represented inclusion or exclusion, or an in between category. Definitions of each of these categories at the indicator level are provided in Online Appendix 1, Table 1-A1 in the supplementary materials.

Of these, 14 indicators measure binary outcomes, with all possible survey responses split into inclusion and exclusion. For these 14 indicators, the ‘in between’ category was used only for the few individuals who refused to answer the relevant survey question.

For the remaining 18 indicators, the ‘in between’ category provides increased nuance regarding the level of inclusion of respondents. This category represents responses that are considered as neither ‘included’ nor ‘excluded’, as well as the responses of those few individuals who refused to answer the relevant survey questions. For example, in the food security indicator, those classified as ‘included’ were food secure, those who were classified as ‘excluded’ experienced severe food insecurity, whilst those in the ‘in between’ category experienced mild-to-moderate food insecurity.

#### Domains

The analysis at the domain level focuses on the proportion of the population from each of the groups that are classified as included, to allow the identification of those domains where significant proportions of each group are not included. Table 4 describes how the cut-offs for the categories of inclusion, exclusion and in between at the domain level are constructed, depending on the number of indicators in each domain.

**TABLE 4:** Definitions of inclusion, exclusion and in between categories for each domain.

No. of indicators in the domain	Domain names	Indicator description	Definition
1	Healthcare access	Healthcare access and quality	Included: Indicator classified as ‘included’
			Excluded: Indicator classified as ‘excluded
			In between: Indicator classified as ‘in between’
2	Economic opportunities and contributions	Labour force status	Included: At least one of the two indicators classified as ‘included’ and the other not classified below ‘in between’ Excluded: At least one of the two indicators classified as ‘excluded’ and the other not classified above ‘in between’ In between: all else
		Public transport availability and affordability	
	Support systems (formal and informal)	Support availability	
		Grant receipt	
	Institutional status	Identity document possession	
		Birth certificate possession	
	Voice	Local decision-making inclusion	
		Voting inclusion	
3	Education	Schooling Basic literacy Basic numeracy	Included: At least two of the three indicators classified as ‘included’ and the other not classified below ‘in between’
			Excluded: At least two of the three indicators classified as ‘excluded’ and the other not classified above ‘in between’
4	Social relationships	Unpaid domestic and care work humiliation Unpaid domestic and care work value Ability to reciprocate support received Community event inclusion	Included: At least three of the four indicators classified as ‘included’ and the other not classified below ‘in between’ Excluded: At least three of the four indicators classified as ‘excluded’ and the other not classified above ‘in between’
	Personal safety	Fuel collection hazards Water collection hazards Safety in the neighbourhood Safety at home	In between: all else
12	Living conditions	Food security Drinking water Domestic water Cooking energy Lighting energy Heating energy Home toilet facilities Toilet modifications Clothing and footwear ownership Clothing and footwear quality Bedding ownership Eviction concern	Included: At least eight of the 12 indicators classified as ‘included’ and the remaining four not classified below ‘in between’ Excluded: At least eight of the 12 indicators classified as ‘excluded’ and the remaining four not classified above ‘in between’ In between: all else

*Source:* IDM South Africa data, prepared by Suich and Schneider

Note: See Online Appendix 1, Table 1-A1 for the indicator-level definitions of ‘inclusion’, ‘exclusion’ and ‘in between’ for each of the indicators.

Given the lack of empirical standards or levels of achievement set in the literature for measuring inclusion, we have set cut-off levels that seem to have good face validity based on qualitative findings in the literature, and although we recognise that these are relatively arbitrary, it is beyond the scope of this article to conduct sensitivity testing on these cut-offs, although further investigation is recommended.

### Ethical considerations

This article has used an existing dataset, and information regarding the original studies’ ethical approval is provided. However, because the analysis presented in this article was conducted using this already existing dataset, no ethical clearance certificates are included in this submission.

The ethical approvals for the original study were gained from the Australian National University Human Research Ethics Committee (Protocol No. 2016/355); South African Human Sciences Research Council Research Ethics Committee (Protocol No. REC 5/21/11/18); Limpopo Provincial Research Ethics Committee (Clearance Certificate No. LPREC/35/2018: PG). No ethical certificates were issued, only approval numbers were recorded.

## Results

### Disability severity and gender disaggregated results

#### Domain results

Table 5 shows the domain results by inclusion category for the six groups, disaggregated by disability severity and gender. The following presentation of results focuses on the proportion in each of these six groups categorised as included, unless specifically stated otherwise.

**TABLE 5:** Domain results by inclusion category, disaggregated by disability severity and gender (% weighted).

Domain	Category	None		Mild		Moderate		*p* adjusted
		Male	Female	Male	Female	Male	Female	
Social relationships	Inclusion	33.5	33.2	28.2	32.3	27.2	28.2	0.22728
	In between	66.5	66.7	71.8	67.6	72.1	71.8	-
	Exclusion	0	0.1	0	0.1	0.7	0	-
Living conditions	Inclusion	70.5	74.2	44.6	53.9	46.1	44.9	NaN†
	In between	29.5	25.8	55.4	46.1	53.9	55.1	-
	Exclusion	0	0	0	0	0	0	-
Economic opportunities and contributions	Inclusion	65.0	61.7	45.4	57.4	46.2	54	0.00006
	In between	25.3	25.8	40.1	28.2	32.1	27.7	-
	Exclusion	9.7	12.4	14.5	14.4	21.7	18.3	-
Support systems (formal and informal)	Inclusion	88.8	89	30.3	40.7	37.9	38.1	0.00006
	In between	11.1	10.9	57.4	47.7	54.5	46.1	-
	Exclusion	0.1	0.1	12.3	11.6	7.5	15.8	-
Institutional status	Inclusion	60.4	63.6	45.4	50.1	47.1	44.1	0.00006
	In between	35.8	32.7	51.7	48.8	52.5	54.3	-
	Exclusion	3.7	3.7	2.9	1.1	0.4	1.6	-
Voice	Inclusion	40.5	46.8	53.8	52.6	45	57.5	0.00006
	In between	27.5	28.4	24.9	30.9	28.6	26.5	-
	Exclusion	32	24.8	21.3	16.5	26.4	16.1	-
Education	Inclusion	62.7	66.8	28.2	38.6	25.1	32.1	0.00006
	In between	32.2	28.4	41.1	38.2	47.8	44.8	-
	Exclusion	5	4.8	30.7	23.2	27.2	23.2	-
Healthcare access	Inclusion	94.3	90.1	85.8	85.5	83.1	85	0.00006
	In between	4.2	9.1	9.2	13.3	14.8	13.7	-
	Exclusion	1.5	0.8	5	1.2	2.1	1.3	-
Personal safety	Inclusion	24.7	13.7	18.8	9.5	22.1	10.2	0.00006
	In between	75.3	86.3	81.2	90.4	77.9	89.2	-
	Exclusion	0	0	0	0.2	0	0.6	-

*Source*: IDM South Africa data, prepared by Suich and Schneider

† , Note that because there are no individuals in the exclusion category in the living conditions domain, no *p*-value can be calculated for this domain.

Overall, there is a pattern of declining inclusion levels with increasing disability severity (from none through mild and moderate), but there is no single pattern in the differences between men and women across the three disability severity classifications. For all domains except social relationships, there are statistically significant and sometimes large differences in the outcomes amongst the three disability severity classifications, although there are relatively low levels of inclusion across all three disability severity classifications for many of the domains.

Healthcare access is the only domain in which a majority of all six groups are categorised as included (all in excess of 80%), and economic opportunities and contributions is the only other domain for which more than 50% of all six groups are classified as included.

The worst outcomes for all three disability severity classifications are in the domains of social relationships and personal safety, with inclusion rates of 50% or less for all six groups. Inclusion rates for the latter domain are less than 25% for all six groups, and the female inclusion gap (the difference in inclusion levels where women have worse outcomes) is the largest, with inclusion rates for women approximately half of those for men in each disability severity classification.

In the education domain, inclusion rates for men are lower than for women across all three disability severity classifications. However, the male inclusion gap (i.e. the difference in inclusion levels where men have worse outcomes) is largest for men with no disabilities and for moderate disabilities in the voice domain, but is largest for men with mild disabilities in the economic opportunities and contributions domain.

For individuals with no disabilities, there are three domains for which inclusion rates are lower for women than men (economic opportunities and contributions, healthcare access and personal safety), and four domains in which inclusion rates are lower for men than women (living conditions, institutional status, voice and education). There are virtually no differences in outcomes between men and women in two domains for those with no disabilities (social relationships and support systems).

For those with mild disabilities, lower inclusion rates for women occur only in the personal safety domain, whilst lower inclusion rates for men occur in five domains (living conditions, economic opportunities and contributions, support systems, institutional status and education), and there are virtually no differences between men and women in three domains (social relationships, voice and healthcare access).

Finally, for those with moderate disabilities, lower inclusion rates for women occur in two domains (personal safety and institutional status), lower inclusion rates for men occur in three domains (economic opportunities and contributions, voice and education), and there is virtually no difference in outcomes in four domains (social relationships, living conditions, support systems and healthcare access).

There are five domains in which those with no disabilities have substantially higher inclusion rates than those with mild and moderate disabilities – living conditions, economic opportunities and contributions, support systems, institutional status and education. There are also smaller but important differences in healthcare access. Those with mild disabilities have higher inclusion rates than those with moderate disabilities for living conditions and education, and the differences between the two groups are relatively smaller for other domains. The voice domain demonstrates an atypical pattern, with individuals with no disabilities less likely to be classified as included compared with individuals with mild or moderate disabilities.

#### Indicator results

The results for each of the indicators, disaggregated for each of the six groups (by disability severity and gender), are shown in Table 6 and, in contrast to the results at the domain level, the discussion focuses on the proportion categorised as excluded – at this level, it may be desirable for policymakers to focus on the very worst outcomes.

**TABLE 6:** Indicator results by inclusion category, disaggregated by disability severity and gender (%, weighted).

Indicator	Category	None		Mild		Moderate		*p* (adjusted)
		Male	Female	Male	Female	Male	Female	
**Social relationships**								
Unpaid domestic and care work humiliation	Included	97.3	97.2	97.1	97.2	96.9	96	1
	In between	0.5	0.4	0	0.3	0	0.4	-
	Excluded	2.2	2.5	2.9	2.6	3.1	3.6	-
Unpaid domestic and care work value	Included	89.6	87.8	89.3	89.4	84.3	91.8	0.27036
	In between	0.3	0.1	0.2	0.3	0.2	0	-
	Excluded	10.2	12.1	10.5	10.3	15.5	8.2	-
Ability to reciprocate support	Included	43.2	42.2	35.1	44	39.4	36.7	0.96132
	In between	0.3	0.6	0	0.5	0.8	0.9	-
	Excluded	56.5	57.3	64.9	55.5	59.8	62.5	-
Community event inclusion	Included	52.6	49.4	60.6	52.5	55.4	44.7	0.00006
	In between	36.7	40.3	26.5	32	27.5	31.3	-
	Excluded	10.7	10.3	12.8	15.5	17.1	24.1	-
**Living conditions**								
Food security	Included	45.8	42.7	26.5	31.4	29.9	23.3	0.00006
	In between	18.2	21.4	20.2	16.3	20.8	16	-
	Excluded	36	35.9	53.3	52.3	49.3	60.7	-
Drinking water	Included	80.9	85.3	69.3	77.7	67.4	72.4	0.00006
	In between	17.2	12.9	25.5	19.5	29.4	23.6	-
	Excluded	1.9	1.8	5.2	2.7	3.2	4	-
Domestic water	Included	82.4	87.1	68.4	79.2	70.7	70.5	0.00006
	In between	17.5	12.8	31.6	20.6	28.8	29.5	-
	Excluded	0.1	0.2	0	0.2	0.6	0	-
Cooking energy	Included	73.5	72.3	63	63	59.8	52	0.00006
	In between	21.8	20.2	28	28.5	36	39.9	-
	Excluded	4.6	7.5	9.1	8.5	4.2	8.2	-
Lighting energy	Included	79.7	82.7	63.9	71.7	61.4	63.1	0.00006
	In between	17	14.7	31.2	25.2	37.5	35.4	-
	Excluded	3.3	2.6	4.9	3	1.1	1.6	-
Heating energy	Included	63.5	60.3	46.7	60.1	44.5	44.5	0.00006
	In between	20.3	22	28.4	24.8	35	32.6	-
	Excluded	16.2	17.6	24.9	15.2	20.5	22.9	-
Home toilet facilities	Included	88.2	84.8	82.3	80.9	90.4	84.1	0.00006
	In between	10	13.5	16.8	17.7	8.2	14.7	-
	Excluded	1.8	1.7	0.9	1.5	1.4	1.2	-
Toilet modifications	Included	98	96.3	53.9	71.7	69.3	75.9	0.00006
	In between	0.4	0.7	13.1	7.6	10.8	5.6	-
	Excluded	1.6	3	33	20.7	19.8	18.5	-
Clothing and footwear ownership	Included	85.4	92.5	72	84.7	69.7	79.3	0.00006
	In between	0.1	0.2	0	0.3	0.7	0	-
	Excluded	14.5	7.4	28	15	29.6	20.7	-
Clothing and footwear quality	Included	69.6	78.4	53.3	63.7	51	56.9	0.00006
	In between	19.4	15.2	31	22.5	32.2	25.2	-
	Excluded	11.1	6.4	15.7	13.8	16.8	17.9	-
Bedding ownership	Included	81.3	80.1	78.9	68.5	69.8	67.1	0.00006
	In between	0.1	0.1	0	0.1	0	0.1	-
	Excluded	18.6	19.8	21.1	31.3	30.2	32.7	-
Eviction concern	Included	86.9	91.6	89.7	88	85.1	87	0.00018
	In between	0.1	0.1	0.4	0.4	0	0	-
	Excluded	13	8.2	9.8	11.6	14.9	13	-
**Economic opportunities and contributions**								
Labour force status	Included	70.8	60.8	73.2	70.1	65.1	63.2	0.00006
	In between	25.6	26.3	15.9	15	13	12.6	-
	Excluded	3.6	12.9	10.9	14.9	21.9	24.2	-
Public transport availability and affordability	Included	41.8	47.7	21.5	36.3	26.3	40	0.00006
	In between	35.9	34.8	39	37.5	39.3	35.2	-
	Excluded	22.3	17.4	39.5	26.2	34.3	24.7	-
**Support systems (formal and informal)**								
Support availability	Included	91.6	90.5	77.3	84	83.6	71.5	0.00006
	In between	0.3	0.4	0.2	0.5	0.3	0.7	-
	Excluded	8.1	9.1	22.5	15.6	16.1	27.8	-
Grant receipt	Included	96.8	98	39.6	42.8	45.9	47.8	0.00006
	In between	0	0	1	2.2	0.6	3.5	-
	Excluded	3.2	2	59.4	55	53.5	48.8	-
**Institutional status**								
Identity document possession	Included	88.5	90.5	90.8	97.4	95.7	96.7	0.00006
	In between	0	0	0	0.2	0	0.1	-
	Excluded	11.4	9.5	9.2	2.4	4.3	3.2	-
Birth certificate possession	Included	66.9	65.5	49.3	47.2	47.7	42.3	0.00006
	In between	1.3	3.9	2.7	4.2	3.2	3.5	-
	Excluded	31.8	30.6	47.9	48.6	49.1	54.2	-
**Voice**								
Local decision-making inclusion	Included	24.5	23.9	42.8	34.2	30.2	32.1	0.00006
	In between	47.1	49.4	30.3	39.1	39.4	44.1	-
	Excluded	28.3	26.7	26.9	26.7	30.4	23.8	-
Voting inclusion	Included	47.7	58	64.9	67.3	53.6	72.1	0.00006
	In between	23.1	21.4	12.8	14.3	16.5	8.7	-
	Excluded	29.3	20.5	22.3	18.4	29.9	19.3	-
**Education**								
Schooling	Included	51.4	52.8	24.7	26.3	22.9	24.8	0.00006
	In between	36.9	35.7	31.3	35.8	36.6	31.8	-
	Excluded	11.7	11.5	44	38	40.5	43.4	-
Basic literacy	Included	70.4	77	41.8	52.8	36	49.7	0.00006
	In between	25.7	18.8	30.3	26.3	36.4	27.4	-
	Excluded	4	4.2	27.8	21	27.6	22.8	-
Basic numeracy	Included	65.3	66.1	35.6	41.9	31.7	39.4	0.00006
	In between	25.7	24.7	33	30.7	35	34	-
	Excluded	9	9.2	31.5	27.4	33.2	26.6	-
**Healthcare access**								
Healthcare access and quality	Included	94.3	90.1	85.8	85.5	83.1	85	0.00006
	In between	4.2	9.1	9.2	13.3	14.8	13.7	-
	Excluded	1.5	0.8	5	1.2	2.1	1.3	-
**Personal safety**								
Fuel collection hazards	Included	96.8	97.9	94.8	98.7	95.7	96.7	0.07536
	In between	0.1	0.1	0.2	0	0	0	-
	Excluded	3.1	2.1	4.9	1.3	4.3	3.3	-
Water collection hazards	Included	97.8	97.6	97.4	98.7	96.5	96.4	1
	In between	0.4	0.4	0	0.2	0.6	0.1	-
	Excluded	1.8	2	2.6	1.1	2.9	3.5	-
Safety at home	Included	27.5	15.1	20.3	10.5	26.8	15.8	0.00006
	In between	69.2	75.2	77.1	75.8	66.5	67.9	-
	Excluded	3.2	9.7	2.6	13.7	6.6	16.3	-
Safety in the neighbourhood	Included	8.6	5.7	4	5.2	11.1	3	0.00006
	In between	74.9	69.6	73.4	66.6	61	52.3	-
	Excluded	16.4	24.6	22.6	28.2	27.9	44.7	-

*Source*: IDM South Africa data, prepared by Suich and Schneider

Three main patterns of differences amongst the three disability severity classifications can be observed across the indicator-level results in Table 6:

1. Exclusion rates are lowest for those with no disabilities, highest for those with moderate disabilities and in between for those with mild disabilities. This is the most common pattern and holds for 13 indicators across six domains.
2. The same or similar exclusion rates are experienced by those with mild and moderate disabilities, and these are worse than for individuals with no disabilities. This pattern holds for eight indicators across four domains.
3. There are no statistically significant differences amongst the three disability severity classifications, which is true for six indicators in three domains.

There are also two indicators in two domains where those with mild disabilities have higher exclusion rates than those with no and moderate disabilities, and three indicators across two domains where those with no disabilities have the highest exclusion rates compared to those with mild and moderate disabilities.

For individuals with no disabilities, there are few differences between men and women for 23 indicators out of 32, men have higher exclusion rates for five indicators and women have higher exclusion rates for four indicators. For individuals with mild disabilities, there are 12 indicators where there are no differences in exclusion rates for men and women, whilst men experience higher exclusion rates than women in 15 indicators and women have higher exclusion rates than men in five indicators. For individuals with moderate disabilities, there are 12 indicators with no gender differences in exclusion rates, higher exclusion rates for men in eight indicators and higher exclusion rates for women in 12 indicators.

The ability to reciprocate indicator has the worst outcomes for all six groups. Other indicators with high exclusion levels amongst all six groups are food security and current possession of a birth certificate. Indeed, these are the only three indicators with exclusion rates of 30% or more of men and women with no disabilities. In addition to these three, very high exclusion rates (i.e. in excess of 50% categorised as excluded) occurred in three indicators for women with mild disabilities, five for men with mild disabilities, six for men with moderate disabilities and four for women with moderate disabilities. Indicators with high exclusion rates common to the four groups of women and men with mild and moderate disabilities are food security, grant receipt and the three indicators in the education domain.

Three of the indicators where the male exclusion gap is largest (i.e. the difference in the proportions categorised as ‘excluded’, where men have worse outcomes) are for voting inclusion, clothing and footwear ownership and public transport availability and affordability, with little variation across the disability severity classifications. However, the largest female exclusion gap (i.e. the difference in the proportions classified as ‘excluded’, where women have worse outcomes) differs with disability severity – for women with no disabilities, the gap is largest for labour force participation, for women with mild disabilities, it is safety at home, and for women with moderate disabilities, it is for safety in the neighbourhood.

### Affect severity domain results

The results presented at the domain level focus on the proportion of each of the four groups (disaggregated by affect severity and gender) categorised as included, unless otherwise stated. There are three main patterns of differences in inclusion levels between the two affect severity classifications (none and moderate) that are identifiable in Table 7, including:

1. No statistically significant differences between affect severity for two domains (social relationships and institutional status), with particularly low inclusion levels for social relationships;
2. Lower inclusion levels for those with moderate affect problems compared to those with none for six domains (living conditions, economic opportunities and contributions, support systems, education, health care access and personal safety), with particularly low inclusion levels for all four groups in education and personal safety;
3. Similar, relatively low, inclusion levels for both affect groups, but lower inclusion amongst men compared to women for the voice domain.

**TABLE 7:** Domain results by inclusion category, disaggregated by affect severity and gender (%, weighted).

Domain	Category	None		Moderate		*p* adjusted
		Male	Female	Male	Female	
Social relationships	Inclusion	33.1	33.9	32.1	27.6	0.01264
	In between	66.9	66.1	67.5	72.2	-
	Exclusion	0	0	0.3	0.2	-
Living conditions	Inclusion	71.6	73.4	48	53.9	Na
	In between	28.4	28.4	52	46.1	-
	Exclusion	0	0	0	0	-
Economic opportunities and contributions	Inclusion	66.3	62	45.2	55	0.00004
	In between	24.9	26	34.7	27.3	-
	Exclusion	8.8	12	20.1	17.7	-
Support systems (formal and informal)	Inclusion	85.9	83.2	69.9	67.2	0.00004
	In between	13.4	15	26.9	27.1	-
	Exclusion	0.7	1.8	3.2	5.7	-
Institutional status	Inclusion	58.7	61.2	61.2	58.3	0.01664
	In between	38.2	35.6	32.5	37.7	-
	Exclusion	3.1	3.2	6.3	4	-
Voice	Inclusion	41.4	49.1	41.5	44.7	0.00004
	In between	27.7	27.1	25.8	34.6	-
	Exclusion	30.9	23.9	32.7	20.8	-
Education	Inclusion	60.6	62.1	51.7	57.4	0.00008
	In between	32.6	30.5	37.8	31.6	-
	Exclusion	6.8	7.4	10.5	10.9	-
Healthcare access	Inclusion	94.5	90.6	87.1	83.6	0.00004
	In between	4.3	8.6	8.6	15.3	-
	Exclusion	1.2	0.8	4.3	1.1	-
Personal safety	Inclusion	25.5	14.5	17.4	6.8	0.00004
	In between	74.5	85.5	82.5	92.9	-
	Exclusion	0	0	0.1	0.2	-

*Source*: IDM South Africa data, prepared by Suich and Schneider

Na, not available.

Healthcare access is the only domain with high inclusion rates for all four groups (as for disability severity), and there are three domains with inclusion rates of 50% or more for all four groups (support systems, institutional status and education). The worst outcomes for all four groups are in the three domains of social relationships, voice and personal safety, with inclusion rates of 50% or less for each of the four groups.

There are three domains for which inclusion rates are lower for women than men for both classifications of affect severity – personal safety (as for disability severity), healthcare access and support systems, and one where inclusion rates for men are lower than for women, the voice domain (as is true for the comparison of disability severity).

The largest inclusion gaps between the two affect classifications are in the three domains of living conditions, support systems and economic opportunities and contributions (in each case, with much worse outcomes for those with moderate affect severity).

For individuals with no affect problems, there were four domains for which there are no inclusion gaps between men and women (social relationships, living conditions, institutional status and education), whilst men have lower inclusion rates than women in one domain (voice) and women had lower inclusion rates than men on four domains (economic opportunities and contributions, support systems, healthcare access and personal safety).

For those with moderate affect problems, there were no differences between men and women for two domains (social relationships and institutional status), men had lower inclusion rates in four domains (living conditions, economic opportunities and contributions, voice and education), whilst women had lower inclusion rates in the remaining three domains (support systems, healthcare access and personal safety). For those with moderate affect difficulties, the largest female inclusion gap was in personal safety, whilst the largest male inclusion gap was in economic opportunities and contributions. The latter result is likely to be related to the number of men and women in the sample size and to the higher proportion of older women (than men) in the group, who are more likely to be not in the labour force by choice. For those with moderate affect difficulties, the inclusion gaps (where they occur) tend to be larger for men, but there are a larger number of domains where men have better outcomes on an average than women.

## Discussion

### Levels of inclusion by disability and affect severity

As can be seen from these results, inclusion levels decline with increasing disability severity, and there are fewer differences in inclusion levels between those with and without affect difficulties than for those with functioning difficulties. The results also demonstrate the universally poor outcomes for social relationships and personal safety and the relatively good outcomes for healthcare access, regardless of gender, disability severity or affect severity.

The healthcare access domain showed the best outcome of all domains with at least 80% of each group being in the ‘included’ category. This is likely partly because of the single indicator covering healthcare use and quality in the domain. The domain does, nevertheless, show the expected decline in inclusion associated with increasing disability severity and affect severity. The high number of older people in the mild and moderate disability severity classifications, many of whom are likely to have chronic health conditions, could reflect the high rate of use of healthcare services by this group. A more nuanced analysis by age could help elucidate this.

The domain-level results also show that men are often less likely to be categorised as included for more indicators within a domain than women in that disability severity classification. This is explained, at least in part, by the smaller number of men with disabilities compared to women – only 34% and 31% of people with mild and moderate disabilities, respectively, are men – in line with international evidence that there are a higher proportion of women than men with disabilities, particularly amongst older age cohorts (Mitra & Sambamoorthi 2014). Thus, a lack of inclusion would have to affect a much larger number of women (absolutely) for the proportions of men and women experiencing that lack of inclusion to be equal.

The comparison of people with functioning disabilities and those with affect difficulties suggests that people with functioning disabilities are less likely to be included across a larger number of domains than those with affect difficulties, particularly for those with moderate disabilities. One exception is in the voice domain where women with moderate affect difficulties show less inclusion than women with moderate functioning disabilities. The higher levels of inclusion amongst those with functioning disabilities in this domain may be linked to the combination of involvement in disabled people’s organisations, and the explicit efforts by the electoral commission and others to improve voting participation by people with disabilities (likely focusing on those with functioning disabilities), where people with psychosocial disability still experience high levels of stigma (Semrau et al. 2015) and could be finding it difficult to have a voice.

The use of a moderate or severe affect as a measure of psychosocial disability has merit but remains to be fully validated. However, the proposed use of a moderate or severe level of affect difficulty as a measure of a ‘seventh’ type of disability as demonstrated here provides important and useful information in understanding differing inclusion levels of those experiencing different types and severity of disabilities.

The use of existing datasets for uses beyond their original design has cost advantages, but also brings a number of limitations and constraints, primarily related to the fact that the required survey questions are not necessarily the same for each purpose. For example, the domain of healthcare access has only one indicator that can be created from the original dataset, but a tool designed specifically to measure and monitor the progressive realisation of disability inclusion would include more questions related to health, including, for example, whether healthcare treatment received was appropriate and/or resulted in improved or stabilised health conditions, about the affordability and accessibility of healthcare, about the need for and use of assistive devices and carers and so on.

Whilst the development of the IDM followed a participatory process in a number of countries to identify the relevant domains and indicators for measuring deprivation, this process did not focus on the identification of domains and indicators of importance in measuring inclusion by people with disabilities (including those with affect difficulties). A similar participatory process involving people with disabilities identifying and prioritising indicators and domains would be necessary to develop tools that covered a comprehensive set of domains and indicators and could accurately measure and monitor inclusion. This participatory process could also be designed to consider what realistic and useful cut-off points should be applied to determine inclusion or exclusion, both at the indicator and domain levels. Finally, such a process could assist in prioritising policy responses, as those seen as most acceptable by people with disabilities could be selected. Such a process would by its very nature and its outcomes foster the development of an inclusive society.

### Prioritising policy responses

One of the major uses of this baseline, albeit partial, is that it enables the identification of the best and worst outcomes across the measured domains for a range of different sub-groups – whether for men or women, those with no, mild or moderate disability severity and those with no or moderate affect difficulties. Domain level results highlight the general areas where levels of inclusion are highest (and where exclusion levels are highest), and the indicator-level data show more detail of the areas that need to be targeted in policy responses. There are several approaches to using the data to design policy responses, each of which results in a different range of domains that may initially be prioritised and targeted. This information could be used by a variety of policymakers, from those within the various levels of government, to those working in the non-government sector and, specifically, those advocating for the progressive realisation of inclusion.

One approach is to target those domains closest to reaching an acceptable level of ‘inclusion’ – those individuals falling just below the inclusion cut-offs are targeted to lift them above the cut-off. In poverty reduction strategies, this is often the most straightforward and cheapest strategy but can result in those farthest from the cut-off – those who are the poorest – being left out of the interventions (Ravallion 2020). This would mean targeting those domains (and the constituent indicators), where inclusion levels are relatively high, and where the majority of remainder are in the ‘in between’ category. For example, for those with affect difficulties, this would involve targeting improvements initially in the support systems and living conditions domains, whilst for those with disabilities policies would initially target the two domains of living conditions and institutional status.

An implication of this strategy is that it is unlikely to dramatically change individuals’ daily experiences – as it would mean that only relatively modest improvements would be achieved for a relatively small number of people and would mean that those who are most excluded would remain highly marginalised, continuing to bear the high costs of this exclusion, in terms of human rights violations and financial outlay.

Another approach is to target those domains farthest from inclusion – prioritising those domains with either the lowest inclusion levels or those with the highest exclusion levels (or both). For example, if a choice were made to prioritise policy responses in those domains where inclusion levels are lowest, the two domains likely to be prioritised for both disability severity and affect severity would be personal safety and social relationships. Policies targeting highest exclusion levels for disability severity would prioritise the domains of education and voice, whilst for affect severity, they would target economic opportunities and contributions and voice.

In contrast to the personal safety and social relationships domains, where there are only very small proportions categorised as excluded at the domain level, the voice domain has both relatively low levels of inclusion and some of the highest exclusion levels for men and women of all disability and affect severity classifications, and worse outcomes for men across both disability and affect severity classifications. The education domain has low levels of inclusion for men and women with mild and moderate disabilities, and the highest exclusion levels for these subgroups, outcomes that are considerably worse than for individuals with no disabilities.

The aim of targeting the most excluded would be to progressively reduce the more extreme levels of exclusion faced by these groups, although a disincentive to policymakers may be the potentially high costs and effort required to bring those most excluded and least included to the required level of inclusion.

Another approach to targeting is to prioritise those with disabilities and target those domains where the gap between those with disabilities and those without is largest, and those with disabilities have the worst outcomes. Groce and Kett (2013) refer to this as the disability and development gap, describing the situation where, when people without disabilities progress in their development, people with disabilities are left behind because of a lack of inclusive policies that ensure necessary accommodation and adaptations for different impairments. For those with disabilities, the two priorities would be the domains of support systems and education, whilst for those with affect difficulties, they would be living conditions and support systems. The focus on inclusive education policies and interventions (as noted in the introduction) can be seen as an attempt to reduce this gap between those with and without disabilities.

A unique feature of this dataset is that it includes those with and without functioning and affect disabilities, which improves our understanding of the domains demonstrating an overall lack of inclusion arising from the high levels of poverty and extreme inequality in South Africa, and also of those domains where a lack of inclusion is related to disability status. Excepting the last approach described above, the selection of policy responses to improve inclusion requires an explicit decision from policymakers about whether to specifically target policy responses to those with functioning disabilities and affect difficulties, or to develop policies aimed at the whole population, for example, where universally poor outcomes are achieved. Even where policymakers aim to increase inclusion across the whole population, different policy responses for different groups may be required to achieve better, more inclusive outcomes amongst people with functioning disabilities and affect difficulties. Developing such targeted responses for different groups and achieving better outcomes for everyone result in a greater equality of opportunities for all.

Being able to disaggregate the data beyond disability or affect severity classifications, for example by gender as has been done in this analysis, has the potential to facilitate even more targeted approaches. These more highly disaggregated data can identify where different responses could be developed where the outcomes of the subgroups differ. For example, there are important differences in the outcomes of men and women in the domains of economic opportunities and contributions and personal safety for those with functioning and affect disabilities, in the institutional status domain for those with mild functioning disabilities, and in the voice domain for those with moderate functioning disabilities.

As can be seen, the domains that are prioritised for targeting are different depending on the choice of policy response approach, a choice which should be made explicitly, and which would strongly benefit from a process of consultation between people with disabilities and policymakers to maximise the effectiveness of responses made.

## Conclusion

Following the proof of concept presented in Schneider & Suich (2021), this article presents a baseline for disability inclusion using data from the IDM survey in South Africa, which could be used in future for monitoring the progressive realisation of disability inclusion within the country, although the data collection tools would benefit from improvements to increase their specificity and relevance to disability inclusion. These results indicate that inclusion levels decline with increasing disability severity and that those with functioning difficulties face greater differences in inclusion levels than those with affect difficulties.

This baseline enables those domains with high, in between or low levels of inclusion and those indicators with high, in between and low levels of exclusion for people with no, mild or moderate disabilities, and people with no or moderate affect difficulties to be highlighted. These results can be used to inform the prioritisation of policy interventions – whether by government or disability support organisations – to improve levels of inclusion for people with disabilities and demonstrate that the targeted domains would differ significantly depending on the approach selected. The final decision on priority targets will benefit from a consultation process involving people with disabilities and all relevant policymakers, a process which would also ideally inform the design of data collection tools specifically for the measurement and monitoring of disability inclusion.
